# A longitudinal cohort study of soil-transmitted helminth infections during the second year of life and associations with reduced long-term cognitive and verbal abilities

**DOI:** 10.1371/journal.pntd.0006688

**Published:** 2018-07-27

**Authors:** Brittany Blouin, Martin Casapia, Lawrence Joseph, Theresa W. Gyorkos

**Affiliations:** 1 Division of Clinical Epidemiology and Centre for Outcomes Research, Research Institute of the McGill University Health Centre, Montreal, Quebec, Canada; 2 Department of Epidemiology, Biostatistics and Occupational Health, McGill University, Montreal, Quebec, Canada; 3 Asociación Civil Selva Amazónica, Iquitos, Perú; Imperial College London, Faculty of Medicine, School of Public Health, UNITED KINGDOM

## Abstract

**Background:**

Soil-transmitted helminth (STH) infection leads to malnutrition and anemia, and has been linked to impaired child development. Previous research on this topic is limited and mostly conducted in school-age children. The goal of this study was to determine the effect of the number of detected STH infections between one and two years of age on subsequent cognitive and verbal abilities, in a cohort of preschool children.

**Methodology/Principal findings:**

A longitudinal cohort study was conducted in 880 children in Iquitos, Peru between September 2011 and July 2016. Children were recruited at one year of age and followed up at 18 months and then annually between two and five years of age. STH infection was measured with the Kato-Katz technique or the direct smear technique. Child development was measured with the Bayley Scales of Infant and Toddler Development-III at the one to three-year visits and with the Wechsler Preschool and Primary Scale of Intelligence-III at the four and five-year visits. Hierarchical multivariable linear regression models were used to account for the repeated outcome measures for each child and Bayesian latent class analysis was used to adjust for STH misclassification. Children found infected with any STH infection between one and two years of age had lower cognitive scores between two and five years of age (between group score differences (95% credible intervals) for infected once, and infected two or three times, compared to never infected: -4.31 (-10.64, -0.14) and -3.70 (-10.11, -0.11), respectively). Similar results were found for *Ascaris* infection and for verbal scores.

**Conclusions/Significance:**

An association was found between having been infected with *Ascaris* or any STH between one and two years of age and lower cognitive and verbal abilities later in childhood. These results suggest that targeting children for STH control as of one year of age is particularly important.

## Author summary

Intestinal worm infections are parasites that can have serious health consequences, including malnutrition. They affect over one billion people in low and middle income countries. It has been proposed that intestinal worm infections can have a negative effect on brain development in young children. This topic, however, has not yet been properly researched. The goal of this study, therefore, was to investigate the effect of worm infections on cognitive and verbal abilities in preschool children between one and five years of age. The study was conducted in the Amazon region of Peru. Children who were infected with these worms between one and two years of age had lower scores on tests of cognitive and verbal abilities between two and five years of age. These results provide empirical evidence suggesting that worm infections may contribute to cognitive impairment in children. Furthermore, they provide support for appropriate control programs targeted to preschool children living in endemic areas and suggest that such programs may lead to improvements in cognitive development.

## Introduction

Investing in child health at a young age is the most effective and efficient strategy to optimize the health of children as they grow. During the first few years of life the brain develops rapidly and small perturbations in this process can have long-term effects on its structural and functional capacity [1]. The early childhood period is therefore considered to be the most important development period across the lifespan [2]. In low and middle-income countries (LMICs), poor child development is alarmingly common with over 200 million children at risk of not reaching their development potential due to the effects of poverty, including malnutrition and inadequate care [1, 3]. Interventions to prevent child malnutrition must occur during the first two years of life to prevent future growth and development deficits [4].

In LMICs, the soil-transmitted helminths (STH), including *Ascaris lumbricoides*, *Trichuris trichiura* and the two hookworm species (*Necator americanus* and *Ancylostoma duodenale*), are a significant contributor to poor health and malnutrition [5]. The burden of disease attributable to STH infections can be even more pronounced when children are exposed to these infections at an early age. Young children are in the most critical period for growth and development across the lifespan and are therefore particularly sensitive to adverse exposures, such as STH infections. Worldwide, approximately 2 billion people are infected with STH infections, of which an estimated 5–10% are children under two years of age [6, 7]. As STH infections are spread through contaminated soil, food and hands, children begin to acquire these infections as they become mobile and begin to explore the environment [8]. *Ascaris* and *Trichuris* are the predominant STH infections in early childhood [9] with infection documented even in the first year of life [10]. Although the World Health Organization (WHO) recommends that children as young as 12 months of age be included in deworming programs in endemic areas, many challenges still need to be overcome to effectively reach this age group in STH control programs [11, 12].

Due to the effects on malnutrition and anemia, STH infections are thought to have an effect on child development. Previous research on this topic has documented associations between STH infections and poor child development; however, the interpretation of this research is limited due to poor research designs [13–21], failure to adjust for important confounding variables [13, 15, 21–23], small sample sizes [15, 22, 24, 25], grouping STH infections with other parasites in the analyses [18, 23] and inadequate and inappropriate statistical analyses [17–22, 24, 25]. Furthermore, no study has previously looked at the long term effect on development of STH infection specifically during the first two years of life—the most critical period for development across the lifespan. The objective of the current research, therefore, was to evaluate the long-term effect of STH infection between one and two years of age on repeated measures of child development between two and five years of age.

## Methods

### Study design and population

The general study methodology has been described in detail elsewhere [26]. Briefly, a longitudinal cohort study was conducted in the rural and peri-urban areas surrounding Iquitos, Peru between September 2011 and July 2016. A total of 1,760 children were recruited at 12 months of age and followed-up at 18 months and at two, three, four and five years of age. During recruitment, a sampling frame for the study was obtained from participating health centre records and from a door-to-door census conducted in the study area by the research team before initiation of the study. The study population consisted of eligible children living in the catchment areas of the twelve major health centres serving the three rural/peri-urban communities surrounding Iquitos (i.e. Nanay, Belén and San Juan), who, during study recruitment, were between 12 and 14 months of age. Study inclusion criteria included: 1) children between 12 and 14 months of age at recruitment; 2) children attending one of the participating health centres for their 12-month routine healthy growth and development visit (note that the parent/guardian of any child who was identified as a potential participant from the sampling frame but who did not attend his/her routine visit at 12 months of age was contacted at home by a research assistant and encouraged to attend); 3) children who were not consulting medical advice for a suspected STH infection; 4) children who had not been dewormed in the six months prior to their recruitment into the study; and 5) children who did not have any serious congenital or chronic medical condition. Study exclusion criteria included: 1) children who lived outside of the identified study area; 2) children whose family planned to move outside of the study area in the year following recruitment; 3) children whose parents did not consent to participate in the study.

### Exposure ascertainment

STH infection was measured at all study visits. At all visits, a parent or guardian of the participating child collected the stool specimen from the child within 24 hours prior to the scheduled study visit. One or two days before the study visit, the research assistant visited the participating child’s parent or guardian in their home and provided the materials and instructions necessary to collect a fresh stool specimen. Stool specimens were transported to the study laboratory where they were analysed by trained technologists. At the 12 and 18 months of age visits, half of the stool specimens were analysed by the Kato-Katz technique within 24 hours of deposition. The remaining stool specimens were stored in 10% formalin and analysed by the direct smear technique after the child had completed their two-year study visit. The rationale for this has been described in detail previously [26]. At all remaining study visits, all stool specimens were analysed with the Kato-Katz technique within 24 hours following deposition. No gold standard technique exists for diagnosing STH infection and all currently available techniques are limited by imperfect sensitivities. Recent sensitivity estimates with 95% credible intervals (CrI) for the Kato-Katz technique include: 63.8% (59.1, 68.6), 82.2% (80.1, 84.5) and 59.5% (56.9, 62.2) for *Ascaris*, *Trichuris* and hookworm infection, respectively [27]. Recent sensitivity estimates with 95% CrIs for the direct smear technique include: 52.1% (46.6, 57.7), 62.8% (56.9, 68.9) and 42.8% (38.3, 48.4) for *Ascaris*, *Trichuris* and hookworm infection, respectively [27]. All children were treated with mebendazole at the two years of age visit and children found STH-infected at subsequent visits were referred to their health centre for treatment.

### Outcome ascertainment

Child development was measured with age-appropriate standardized scales at the one, two, three, four and five years of age study visits (child development was not measured at the 18-month visit). Because no single high quality test exists to measure child development over this entire age range, two different scales were used. At the one to three years of age visits, the cognitive, receptive language, expressive language and fine motor subtests of the Bayley Scales of Infant and Toddler Development, Third Edition (Bayley-III) were used. The Bayley-III subtests were administered, at the one and two-year visits, to all children; and at the three-year visit, for feasibility reasons, to a random sample of 880 children of the original study population. In the event that a child who was randomly sampled to be administered the Bayley-III was lost to follow-up at the three-year visit, the next child scheduled to pass their study visit from the same health centre catchment area, who had not originally been randomly selected, was selected to be administered the Bayley-III. The Bayley-III, which was developed for a US population, required some adaption for use in this setting. A local psychologist with experience using this scale in Peru worked with the research team to adapt some of the words and pictures from the scale to ensure that they were appropriate for use in the study setting. Careful consideration was made to ensure that all adaptations remained age-appropriate. The adapted scale was extensively pre-tested in the area before initiation of the study.

At the four and five years of age visits, the Spanish version of the Wechsler Preschool and Primary Scale of Intelligence, Third Edition (WPPSI-III) was used. The seven subscales used to generate the full scale IQ score, the verbal IQ score and the performance IQ score were administered to the same children randomly sampled to be administered the Bayley-III at the three-year study visit. These subscales include: vocabulary, information, word reasoning, matrices, picture concepts, block design and coding. The WPPSI-III Spanish version has been validated in a Mexican population and was found to be culturally appropriate for this study population (i.e. no adaptation was necessary).

Both scales were administered by highly trained research assistants who had previous healthcare experience (e.g. nurses or nurse-midwives) with a minimum Bachelor’s degree education. Research assistants were unaware of the child’s STH infection status during the assessments. The scales were administrated in secluded areas to avoid distractions and, in the event that a child did not appear to be performing at his/her optimal ability, either a break was taken or the assessment was postponed to a future date. Both scales provide age-scaled composite scores for cognitive ability (i.e. the cognitive composite score from the Bayley-III and the performance IQ score from the WPPSI-III) and verbal ability (i.e. the language composite score from the Bayley-III and the verbal IQ score from the WPPSI-III) and were scored according to the specific instructions provided in the scoring manuals [28, 29]. Raw scores from the four subscales of the Bayley-III and the seven subscales of the WPPSI-III were converted to age-scaled scores according to the exact age of each child at the time they completed the scale. Scaled cognitive scores were generated using the scaled cognitive score from the Bayley-III and by summing the block design, matrices and picture concepts scaled scores from the WPPSI-III. Scaled verbal scores were generated by summing the expressive language and receptive language scaled scores from the Bayley-III and by summing the information, vocabulary and word reasoning scaled scores from the WPPSI-III. Cognitive and verbal scaled scores from both scales were converted to cognitive and verbal composite scores using conversion tables provided in the two manuals. These composite scores have a reference mean of 100 and standard deviation of 15.

### Measurement of covariates

Child height and weight were measured at all study visits. Children were measured without clothes or shoes. Weight was measured with a portable electronic scale accurate to the nearest 0.01 kg in the sitting position for children under two years of age and in the standing position for children two years and older. Length/height was measured using a portable stadiometer accurate to the nearest millimeter. Child length (recumbent position) was measured for children two years of age and younger, and child height (standing position) was measured for children older than two years. A questionnaire was administered to the parent or guardian of participating children at all study visits regarding relevant socio-demographic information and health and medical history.

### Ethics

The study was conducted according to the principles of the Declaration of Helsinki. The study was approved by the Universidad Peruana Cayetano Heredia in Lima, Peru (11005, 12009); the Instituto Nacional de Salud in Lima, Peru (032–11); and, the Research Ethics Boards of the McGill University Health Centre in Montreal, Canada (10-242-PED, 12-026-PED). Written informed consent was obtained from parents/guardians of the participating children. In the event that a parent of the participating child was under the age of 18, written assent was obtained from the minor parent, with written informed consent obtained from their parent or guardian over 18 years of age.

### Statistical analyses

Anonymized data were stored on password-protected computers in locked offices. Double data entry was performed for all entered data and data cleaning followed a comparison of the two databases. Summary statistics of the study population include means and standard deviations for continuous variables and counts and proportions for binary and categorical data.

#### Analyses without adjustment for STH misclassification

Hierarchical linear regression models were used to investigate the effect of the number of times a child was detected STH-infected between one and two years of age on repeated measures of development scores at two, three, four and five years of age. The effect of *Ascaris* infection, *Trichuris* infection and being infected with any of the three STH species (any STH infection) on both cognitive and verbal scores was estimated. The specific effect of hookworm infection was not assessed due to the very low prevalence of hookworm in this age group. The exposure was categorized into found infected zero times, one time, two times and three times. Due to the low number of children who were found infected at all three time points between one and two years of age, the categories for being found infected two and three times were combined.

#### Model selection

Both univariable and multivariable regression models are presented. The covariates included in the final models were chosen based on theoretical knowledge (i.e. confounding variables that are thought to be associated with both the exposure and outcome of interest without being mediators of this relationship) and by statistical criteria. Baseline variables considered as potential confounders included: socioeconomic status (i.e. residential district, urban/rural status, mother’s marital status (i.e. married/common-law vs single), maternal education (i.e. secondary education completed), mother employed, father or mother’s partner employed, number of people living in the home, house material, cooks using gas, presence of electricity in the home, working radio ownership, working television ownership, water source, has a toilet with water and connection to public sewage in the home, and household income); sex; healthcare seeking behavior (i.e. number of healthy growth visits attended from birth to one year of age and vaccines up to date at baseline); hygiene (i.e. number of baths per day and use of soap for bathing); hospitalizations since birth; anthropometry/malnutrition (i.e. stunted, underweight, wasted, birth weight); baseline development scores (i.e. Bayley-III cognitive raw score, Bayley-III receptive language raw score, Bayley-III expressive language raw score and, Bayley-III fine motor raw score); and breastfeeding (i.e. exclusively breastfed to six months and continued breastfeeding at one year). To perform model selection, univariable hierarchical linear regression models were used to determine if each variable was associated with the outcome variables and univariable multinomial regression models were used to determine if each variable was associated with the exposure variables. Correlations and 2 x 2 tables were also used to observe relationships between the confounding variables. The final presented models include confounding variables that are associated with both the exposure and outcome and that affected the association between the exposure and outcome of interest. These include socioeconomic status (i.e. maternal education, cooks using gas, has a toilet with water and connection to public sewage in the home), baseline nutritional status (i.e. stunted), use of health care (i.e. number of healthy growth visits attended from birth to one year of age), baseline development scores (i.e. Bayley-III cognition raw scores) and age.

An individualized intercept term was used to allow each child’s baseline development score to be different and an individualized slope for age was used to allow children to develop at different rates. The age variable was centered to reduce correlation between the individualized intercept and slope.

#### Missing data

Missing exposure and outcome data were imputed using multiple imputation. No covariate had missing data. Multinomial regression models were used as the imputation models for cumulative STH infections and linear regression models were used as the imputation models for development scores. All covariates included in the outcome models were also included in the imputation models as well as other relevant covariates, with complete data, that predicted the missing data, as appropriate. Analyses without adjustment for misclassification and model selection procedures were performed in Stata version 13.1.

#### Analyses with adjustment for STH misclassification

To address exposure misclassification due to imperfect sensitivities and specificities of the diagnostic tests used to measure STH infection, Bayesian latent class hierarchical regression models were used. This method was particularly useful in this context because it allows for individual variation in sensitivity and specificity values (here, due to the fact that, while the majority of stool specimens were analyzed with the Kato-Katz technique, some stool specimens were analyzed with the direct smear technique) and because a gold standard diagnostic technique for STH infection does not exist and therefore the sensitivity and specificity values are not exactly known. This method has been described in detail and used previously [30]. Briefly, within the latent class analysis, three separate models were specified: 1) **The outcome model** is a hierarchical linear regression that models child development scores conditional on latent STH infection (i.e. the true, unmeasured exposure) and the confounding variables mentioned previously. An individualized intercept and slope for age was included according to the previous description. The main effect estimate of interest is the effect of latent STH infection on child development, estimated in this model.; 2) **The exposure models** are logistic regressions that model true latent STH infection status at 12, 18 and 24 months of age, conditional on covariates that predict species-specific STH infection. These covariates were chosen based on Bayesian information criterion (BIC). The exposure models allow for differences in the probabilities of being STH-infected between various groups of children to be accounted for; and 3) **The misclassification models** predict the measured STH infection status at each of the three time points according to the true latent STH infection status at each time point and the sensitivity and specificity values of the diagnostic technique used (i.e. Kato-Katz technique or direct smear technique) at each time point, according to:

$$P({mSTH}_{i}=1)=\begin{array}{c} S_{KK}({tSTH}_{i})({KK}_{i})+(1-C_{KK})(1-{tSTH}_{i})({KK}_{i})+\\ S_{DS}(t{STH}_{i})(1-{KK}_{i})+(1-C_{DS})(1-t{STH}_{i})(1-{KK}_{i}) \end{array}$$

Where:

*i* = study time points

*mSTH*_*i*_ = Measured STH infection status at time point *i* (dichotomous variable)

*tSTH*_*i*_ = Latent, true STH infection status at time point *i* (dichotomous variable)

*KK*_*i*_ = Indicator variable indicating technique used to analyze stool specimen at time point *i*

(*KK*_*i*_ = 1 represents use of the Kato-Katz technique; *KK*_*i*_ = 0 represents use of the direct smear technique)

S_KK_ = Sensitivity of the Kato-Katz technique

C_KK_ = Specificity of the Kato-Katz technique

S_DS_ = Sensitivity of the direct smear technique

C_DS_ = Specificity of the direct smear technique

The exposure and measurement models are used to adjust for STH misclassification and the adjusted STH status is used in the outcome model. Diffuse, non-informative priors were used for all regression coefficients in the outcome and exposure models. Informative priors were specified for the sensitivity and specificity values of the two diagnostic techniques (Table 1). Clinical priors representing the best summary of the information from the published literature and expert opinion [27] as well as optimistic and pessimistic priors were specified for sensitivity analyses. Optimistic priors are those assuming higher accuracy for the techniques than the “best estimate” clinical priors would suggest, and similarly, pessimistic priors assume lower accuracy than the clinical priors would suggest. All prior densities on the sensitivity and specificity parameters were assumed to follow a beta distribution. Although unrealistic, for comparison purposes, analyses were also run assuming that the sensitivity and specificity values were 100%. The models were jointly estimated using the Gibbs sampler, such that the latent true exposure was essentially imputed within the misclassification and exposure models, analogous to missing data techniques, and subsequently included in the outcome model. For the analyses adjusted for misclassification, WinBUGS (version 1.4.3, MRC, Cambridge) was used to run the MCMC process used for all inferences.

**Table 1:** Probability ranges and corresponding coefficients of the beta prior densities for the sensitivities and specificities of the Kato-Katz and direct smear techniques used in the Bayesian latent class analyses to adjust for misclassification of STH infection.

		Clinical priors			Optimistic priors			Pessimistic priors		
		Range	Beta distribution coefficients		Range	Beta distribution coefficients		Range	Beta distribution coefficients	
			α	β		α	β		α	β
**Kato-Katz**	*Ascaris*:									
	Sensitivity	0.55–0.75	55.86	29.63	0.70–0.80	214.34	70.81	0.50–0.60	208.45	170.38
	Specificity	0.95–0.99	231.95	6.26	0.95–0.99	231.95	6.26	0.95–0.99	231.95	6.26
	*Trichuris*:									
	Sensitivity	0.75–0.90	77.85	15.75	0.85–0.95	116.06	12.05	0.70–0.80	214.34	70.81
	Specificity	0.95–0.99	231.95	6.26	0.95–0.99	231.95	6.26	0.95–0.99	231.95	6.26
	Hookworm:									
	Sensitivity	0.52–0.68	85.65	56.78	0.63–0.73	226.28	105.98	0.47–0.57	198.73	183.37
	Specificity	0.95–0.99	231.95	6.26	0.95–0.99	231.95	6.26	0.95–0.99	231.95	6.26
**Direct smear**	*Ascaris*:									
	Sensitivity	0.40–0.60	47.30	47.30	0.55–0.65	220.49	146.68	0.35–0.45	146.68	220.49
	Specificity	0.95–0.99	231.95	6.26	0.95–0.99	231.95	6.26	0.95–0.99	231.95	6.26
	*Trichuris*:									
	Sensitivity	0.55–0.75	55.86	29.63	0.70–0.80	214.34	70.81	0.50–0.60	208.45	170.38
	Specificity	0.95–0.99	231.95	6.26	0.95–0.99	231.95	6.26	0.95–0.99	231.95	6.26
	Hookworm:									
	Sensitivity	0.35–0.55	42.02	51.57	0.50–0.60	208.45	170.38	0.30–0.40	121.41	226.29
	Specificity	0.95–0.99	231.95	6.26	0.95–0.99	231.95	6.26	0.95–0.99	231.95	6.26

### Sample size

A sample size of 880 children with four measures of the outcome per child (i.e. at 2, 3, 4 and 5 years of age) was available for analysis. The primary outcome, cognitive score, was compared between children who were never found STH-infected to children who were found infected one time and two or three times. No estimate of the intraclass coefficient (ICC) for repeated measures of cognitive scores of children between two and five years of age was found in the literature. Therefore, based on preliminary data, an ICC for cognitive scores was estimated to be 0.2. This corresponds to a design effect of 1.6 (design effect = 1 + (# observations per child– 1) × ICC). Assuming that the repeated observations were independent, a total sample size of 3,520 would be available (880 participants × 4 outcome measures per participant = 3,520 outcome measurements). Taking the correlation between repeated measures into account, the effective sample size is 2,200 (3,520 outcome measurements / 1.6 (the design effect) = 2,200). Based on expert opinion in child development, a difference in mean scores of 5 points (i.e. 1/3 of a standard deviation) was considered the minimum clinically significant effect size. Assuming that the standard deviation of cognitive scores is 15, and having known from preliminary data that 43% of the population is unexposed (never STH-infected) and that 36% and 21% of the population were found STH-infected one, and two or three times, respectively (based on preliminary data), an effective sample size of 2,200 would be able to detect a difference of 5 points in cognitive scores between never infected and infected once, and between never infected and infected two or three times, with total 95% confidence interval widths of 2.84 and 3.34, respectively. Therefore, the sample size provided sufficient precision for the planned comparisons.

## Results

### Descriptive statistics

**Fig 1:**
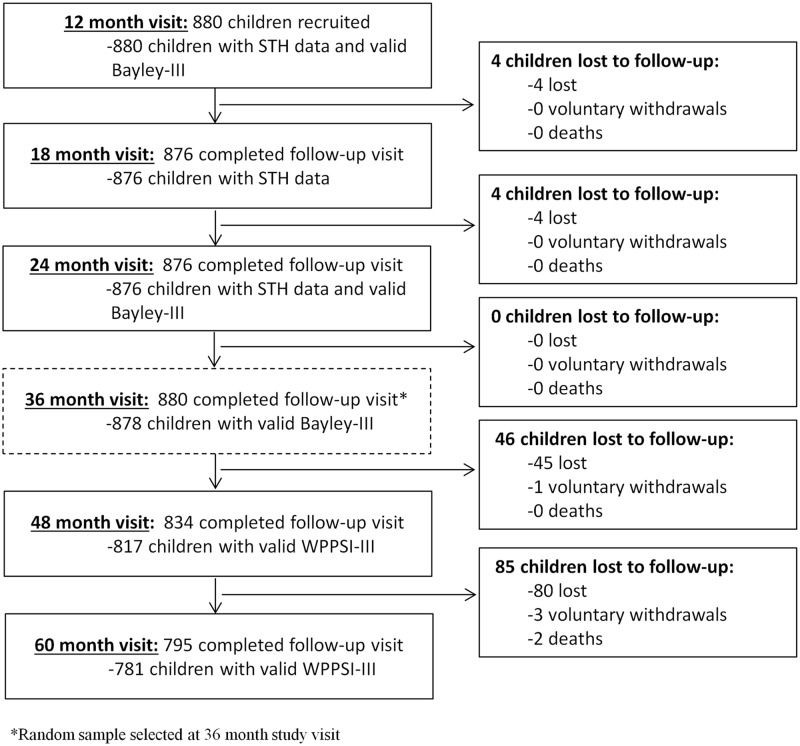
Study flowchart for 880 children randomly sampled at the 36-month visit to be included in this analysis in Iquitos, Peru September 2011 to July 2016.

A description of the population included in this analysis at baseline, including the relationship between the original study population and the random sample, has been described previously [26]. The mean age at recruitment was 12.1 (± 0.28) months with 51.9% of the population being male. At baseline, malnutrition was present with 24%, 7.5% and 1.7% of the population considered to be stunted, underweight and wasted, respectively. The study flowchart is shown in Fig 1. Missing STH infection data were present for four children at 18 months of age and for four children at 24 months of age due to loss to follow-up. Number of times found STH infected between one and two years of age was therefore missing for a total of eight children (< 1%). Development scores were missing for a total of four children at the two years of age visit due to loss to follow-up. At the three years of age visit, development scores were missing for two children due to a protocol violation (two children who should have been administered the Bayley-III were not, due to an error by the research assistant). At the four years of age visit, development scores were missing for a total of 63 children (7%): 46 due to loss to follow-up, 15 due to invalid WPPSI-III measurements (WPPSI-III measurements are considered invalid if a participant scores 0 on two or more performance subscales and/or verbal subscales) and two due to protocol violations. At the five-year visit, development scores were missing for a total of 99 children (11%): 85 due to loss to follow-up and 14 due to invalid WPPSI-III measurements. The majority of children who were lost to follow-up throughout the study moved homes between study visits and could not be located.

Tables 2 and 3 show the detected STH prevalence and intensity data at the 12 months, 18 months and 24 months of age study visits (unadjusted for misclassification). At one year of age the prevalence of being infected with at least one STH infection was 12.4%. This more than tripled by two years of age at which point the prevalence reached 41.1% (Table 2). *Ascaris* was the most common species with 46.6% of the population infected with *Ascaris* at least one time between one and two years of age (Table 3). Hookworm infection was uncommon with only 2% of the population harbouring a hookworm infection at some point between one and two years of age (Table 3). Raw, scaled and composite development scores from the Bayley-III and the WPPSI-III between one and five years of age are presented in Table 4. Overall, composite scores decreased over time with the highest scores obtained at the baseline visit and the lowest scores obtained at the final, five years of age visit.

**Table 2:** STH prevalence and intensity at the 12, 18 and 24 months of age study visits in preschool children in Iquitos, Peru, September 2011 to July 2016.

	12 months^a^	18 months^b^	24 months^c^
*Ascaris*			
Prevalence [% (n)]	11.0 (97)	23.3 (204)^e^	30.7 (269)^e^
Intensity (epg^g^) [mean (sd)]	329.9 (1,416.6)^d^	1,468.3 (7,661.6)^f^	2,304.2 (11,987.5)^e^
Prevalence of moderate/ heavy intensity infection [% (n)]	2.0 (9)^d^	5.6 (24) ^f^	9.6 (84)^e^
*Trichuris*			
Prevalence [% (n)]	2.5 (22)	7.7 (67) ^e^	21.8 (191)^e^
Intensity (epg^g^) [mean (sd)]	26.5 (261.3)^d^	45.0 (346.5) ^f^	46.7 (225.5)^e^
Prevalence of moderate/ heavy intensity infection [% (n)]	0.7 (3)^d^	0.7 (3) ^f^	0.7 (6)^e^
Hookworm			
Prevalence [% (n)]	0.34 (3)	0.57 (5) ^e^	1.37 (12)^e^
Intensity (epg^g^) [mean (sd)]	1.7 (26.8)^d^	3.6 (48.3) ^f^	1.4 (17.6)^e^
Prevalence of moderate/ heavy intensity infection [% (n)]	0 (0)^d^	0 (0) ^f^	0 (0)^e^
Any STH			
Prevalence [% (n)]	12.4 (109)	27.9 (244) ^e^	41.1 (360)^e^

^a^ At the one year of age visit, 449 (51%) stool specimens were analyzed using the Kato-Katz technique and 431 (49%) stool specimens were analyzed using the direct smear technique

^b^ At the 18 months of age visit, 428 (48.9%) stool specimens were analyzed using the Kato-Katz technique and 448 (51.1%) stool specimens were analyzed using the direct smear technique

^c^ At the 24 months of age visit, all available stool specimens were analyzed using the Kato-Katz technique

^d^ Data available for 449 participants (who had their stool specimen analyzed with the Kato-Katz technique)

^e^ Data available for 876 participants

^f^ Data available for 428 participants (who had their stool specimen analyzed with the Kato-Katz technique)

^g^ Eggs per gram of stool

**Table 3:** Frequency of the number of detected STH infections between 12 and 24 months of age for the 880 children included in this analysis*, Iquitos, Peru, September 2011 to July 2016.

	*Ascaris*	*Trichuris*	Hookworm	Any STH
No detected infections [% (n)]	53.4 (466)	74.0 (645)	97.9 (854)	44.5 (388)
One detected infection [% (n)]	30.4 (265)	20.4 (178)	1.8 (16)	33.5 (292)
Two detected infections [% (n)]	13.7 (119)	5.4 (47)	0.2 (2)	18.1 (158)
Three detected infections [% (n)]	2.5 (22)	0.2 (2)	0 (0)	3.9 (34)

*Complete data available for 872 participants

**Table 4:** Raw, scaled and composite scores from the Bayley Scales of Infant and Toddler Development (Bayley-III) and the Wechsler Preschool and Primary Scale of Intelligence (WPPSI-III) at the one to five years of age study visits, Iquitos, Peru, September 2011 to July 2016.

	1 year	2 years^a^	3 years^b^	4 years^c^	5 years^d^
Bayley-III: Cognitive					
Raw [mean (sd)]	42.6 (3.0)	59.2 (3.2)	67.5 (3.1)	NA	NA
Scaled [mean (sd)]	11.0 (1.9)	8.1 (1.3)	7.4 (0.8)	NA	NA
Bayley-III: Receptive language					
Raw [mean (sd)]	13.0 (1.6)	23.9 (2.3)	31.6 (3.4)	NA	NA
Scaled [mean (sd)]	7.9 (1.9)	8.4 (1.2)	8.5 (1.3)	NA	NA
Bayley-III: Expressive language					
Raw [mean (sd)]	13.5 (2.2)	24.7 (3.4)	34.1 (3.8)	NA	NA
Scaled [mean (sd)]	8.9 (1.8)	7.4 (1.5)	7.9 (1.3)	NA	NA
Bayley-III: Fine Motor					
Raw [mean (sd)]	29.2 (1.5)	39.4 (2.1)	47.9 (3.3)	NA	NA
Scaled [mean (sd)]	10.0 (1.6)	10.3 (1.7)	9.6 (1.6)	NA	NA
WPPSI-III: Block design					
Raw [mean (sd)]	NA	NA	NA	18.0 (2.3)	20.1 (2.8)
Scaled [mean (sd)]	NA	NA	NA	10.0 (1.2)	8.1 (1.5)
WPPSI-III: Information					
Raw [mean (sd)]	NA	NA	NA	15.2 (3.9)	18.4 (3.7)
Scaled [mean (sd)]	NA	NA	NA	7.0 (2.5)	6.6 (2.4)
WPPSI-III: Matrices					
Raw [mean (sd)]	NA	NA	NA	3.7 (3.2)	7.5 (4.7)
Scaled [mean (sd)]	NA	NA	NA	6.4 (2.7)	6.7 (3.3)
WPPSI-III: Vocabulary					
Raw [mean (sd)]	NA	NA	NA	7.2 (4.3)	11.0 (4.8)
Scaled [mean (sd)]	NA	NA	NA	6.7 (2.9)	6.8 (2.6)
WPPSI-III: Picture concepts					
Raw [mean (sd)]	NA	NA	NA	4.1 (2.5)	6.0 (3.8)
Scaled [mean (sd)]	NA	NA	NA	7.7 (2.1)	6.3 (2.4)
WPPSI-III: Word reasoning					
Raw [mean (sd)]	NA	NA	NA	2.1 (2.3)	4.3 (3.7)
Scaled [mean (sd)]	NA	NA	NA	6.3 (1.7)	5.4 (2.1)
WPPSI-III: Coding					
Raw [mean (sd)]	NA	NA	NA	4.6 (5.1)	13.7 (6.2)
Scaled [mean (sd)]	NA	NA	NA	6.8 (2.2)	7.5 (2.0)
**Verbal composite score** [mean (sd)]	90.7 (9.0)	87.9 (6.6)	89.5 (6.4)	79.7 (11.9)	77.0 (11.8)
**Cognitive composite score** [mean (sd)]	105.1 (9.7)	90.5 (6.6)	86.8 (4.0)	87.4 (9.5)	81.1 (11.9)

^a^ Missing data for 4 participants

^b^ Missing data for 2 participants

^c^ Missing data for 63 participants

^d^ Missing data for 99 participants

NA: Not applicable (scale was not administered at this study time point)

### Results without adjustment for STH misclassification

Results from univariable and multivariable hierarchical linear regression models for the effects of STH infection (without adjustment for misclassification) between one and two years of age on cognitive and verbal scores between two and five years of age are shown in Tables 5 and 6, respectively. In univariable analyses, children found infected with *Ascaris*, *Trichuris* and any STH infection had cognitive scores between 1 and 2 points lower, on average, compared to children who were never found infected (Table 5). Children found infected with *Ascaris*, *Trichuris* and any STH infection had verbal scores between 2 and 3 points lower, on average, compared to children who were never found infected (Table 6). In multivariable analyses, adjusted for relevant confounding variables, results were attenuated. The effects (beta (95% confidence interval)) on cognitive scores of being infected with any STH infection one time and two or three times between one and two years of age, compared to never being detected infected were: -0.54 (-1.31, 0.23) and -0.52 (-1.41, 0.37), respectively. The same effects on verbal scores were -0.95 (-1.91, 0.01) and -0.75 (-1.87, 0.37), respectively.

**Table 5:** Univariable and multivariable linear regression results for the effect of cumulative *Ascaris* infection, cumulative *Trichuris* infection, and cumulative any STH infection on cognitive scores in preschool children in Iquitos, Peru, September 2011 to July 2016.

	Univariable	Multivariable with multiple imputation ^b^
	β (95% CI)	β (95% CI)
**# times found infected with *Ascaris*:**		
0	REF ^a^	REF
1	-1.45 (-2.29, -0.61) ^a^	-0.64 (-1.40, 0.12)
2–3	-1.98 (-3.03, -0.94) ^a^	-0.63 (-1.59, 0.32)
Mother completed secondary school	4.03 (3.29, 4.78)	2.89 (2.14, 3.64)
Cooks using gas	2.75 (1.97, 3.53)	1.22 (0.46, 1.98)
Toilet with water and connection to public sewage in the home	2.43 (1.69, 3.16)	0.78 (0.06, 1.51)
Stunting at 1 year	-2.49 (-3.34, -1.64)	-1.43 (-2.22, -0.64)
Bayley-III^c^: cognition raw score at 1 year	0.10 (0.06, 0.14)	0.06 (0.02, 0.09)
# healthy growth visits from birth to 1 year of age	0.30 (0.20, 0.40)	0.15 (0.05, 0.25)
Age	-2.76 (-3.04, -2.49)	-2.75 (-3.02, -2.48)
**# times found infected with *Trichuris*:**		
0	REF ^a^	REF
1	-1.07 (-1.99, -0.15) ^a^	-0.14 (-0.99, 0.71)
2–3	-1.94 (-3.55, -0.33) ^a^	-0.30 (-1.76, 1.17)
Mother completed secondary school	4.03 (3.29, 4.78)	2.88 (2.12, 3.64)
Cooks using gas	2.75 (1.97, 3.53)	1.26 (0.50, 2.03)
Toilet with water and connection to public sewage in the home	2.43 (1.69, 3.16)	0.83 (0.10, 1.57)
Stunting at 1 year	-2.49 (-3.34, -1.64)	-1.43 (-2.23, -0.63)
Bayley-III^c^: cognition raw score at 1 year	0.10 (0.06, 0.14)	0.06 (0.02, 0.09)
# healthy growth visits from birth to 1 year of age	0.30 (0.20, 0.40)	0.14 (0.05, 0.24)
Age	-2.76 (-3.04, -2.49)	-2.74 (-3.02, -2.47)
**# times found infected with any STH:**		
0	REF ^a^	REF
1	-1.46 (-2.31, -0.62) ^a^	-0.54 (-1.31, 0.23)
2–3	-1.98 (-2.94, -1.02) ^a^	-0.52 (-1.41, 0.37)
Mother completed secondary school	4.03 (3.29, 4.78)	2.86 (2.11, 3.61)
Cooks using gas	2.75 (1.97, 3.53)	1.26 (0.50, 2.03)
Toilet with water and connection to public sewage in the home	2.43 (1.69, 3.16)	0.76 (0.03, 1.49)
Stunting at 1 year	-2.49 (-3.34, -1.64)	-1.42 (-2.21, -0.63)
Bayley-III^c^: cognition raw score at 1 year	0.10 (0.06, 0.14)	0.06 (0.02, 0.09)
# healthy growth visits from birth to 1 year of age	0.30 (0.20, 0.40)	0.15 (0.05, 0.24)
Age	-2.76 (-3.04, -2.49)	-2.73 (-3.00, -2.46)

^a^ 872 participants included in the analysis (number of times found infected with any STH infection and species-specific infections were missing for 8 participants)

^b^ 880 participants were included in the analyses (complete data available for 754 participants; number of times found infected with any STH infection and species-specific infections was imputed for 8 participants; and cognitive score was imputed for 4, 2, 63 and 99 participants at the 2, 3, 4 and 5-year visit, respectively)

^c^ Bayley III: Bayley Scales of Infant and Toddler Development, Third Edition

**Table 6:** Univariable and multivariable linear regression results for the effect of cumulative *Ascaris* infection, cumulative *Trichuris* infection, and cumulative any STH infection on verbal scores in preschool children in Iquitos, Peru, September 2011 to July 2016.

	Univariable	Multivariable with multiple imputation ^b^
	β (95% CI)	β (95% CI)
**# times found infected with *Ascaris*:**		
0	REF ^a^	REF
1	-2.03 (-3.10, -0.96) ^a^	-0.92 (-1.88, 0.03)
2–3	-2.10 (-3.43, -0.77) ^a^	-0.20 (-1.40, 1.00)
Mother completed secondary school	4.77 (3.81, 5.73)	2.92 (1.98, 3.85)
Cooks using gas	3.69 (2.69, 4.68)	1.61 (0.65, 2.57)
Toilet with water and connection to public sewage in the home	3.74 (2.82, 4.67)	1.64 (0.73, 2.55)
Stunting at 1 year	-3.71 (-4.80, -2.63)	-2.31 (-3.30, -1.32)
Bayley-III^c^: cognition raw score at 1 year	0.16 (0.11, 0.20)	0.09 (0.05, 0.14)
# healthy growth visits from birth to 1 year of age	0.48 (0.35, 0.61)	0.27 (0.15, 0.39)
Age	-4.28 (-4.55, -4.01)	-4.25 (-4.52, -3.98)
**# times found infected with *Trichuris*:**		
0	REF ^a^	REF
1	-2.07 (-3.24, -0.90) ^a^	-0.64 (-1.69, 0.41)
2–3	-2.77 (-4.81, -0.72) ^a^	-0.24 (-2.09, 1.60)
Mother completed secondary school	4.77 (3.81, 5.73)	2.87 (1.93, 3.82)
Cooks using gas	3.69 (2.69, 4.68)	1.61 (0.64, 2.57)
Toilet with water and connection to public sewage in the home	3.74 (2.82, 4.67)	1.66 (0.74, 2.58)
Stunting at 1 year	-3.71 (-4.80, -2.63)	-2.28 (-3.28, -1.28)
Bayley-III^c^: cognition raw score at 1 year	0.16 (0.11, 0.20)	0.10 (0.05, 0.14)
# healthy growth visits from birth to 1 year of age	0.48 (0.35, 0.61)	0.27 (0.15, 0.39)
Age	-4.28 (-4.55, -4.01)	-4.25 (-4.52, -3.97)
**# times found infected with any STH:**		
0	REF ^a^	REF
1	-2.24 (-3.31, -1.17) ^a^	-0.95 (-1.91, 0.01)
2–3	-2.88 (-4.10, -1.66) ^a^	-0.75 (-1.87, 0.37)
Mother completed secondary school	4.77 (3.81, 5.73)	2.89 (1.95, 3.84)
Cooks using gas	3.69 (2.69, 4.68)	1.63 (0.67, 2.60)
Toilet with water and connection to public sewage in the home	3.74 (2.82, 4.67)	1.58 (0.67, 2.50)
Stunting at 1 year	-3.71 (-4.80, -2.63)	-2.29 (-3.28, -1.29)
Bayley-III^c^: cognition raw score at 1 year	0.16 (0.11, 0.20)	0.09 (0.05, 0.14)
# healthy growth visits from birth to 1 year of age	0.48 (0.35, 0.61)	0.27 (0.15, 0.39)
Age	-4.28 (-4.55, -4.01)	-4.25 (-4.52, -3.97)

^a^ 872 participants included in the analysis (number of times found infected with any STH infection and species-specific infections were missing for 8 participants)

^b^ 880 participants were included in the analyses (complete data available for 754 participants; number of times found infected with any STH infection and species-specific infections was imputed for 8 participants; and verbal score was imputed for 4, 2, 63 and 99 participants at the 2, 3, 4 and 5-year visit, respectively)

^c^ Bayley III: Bayley Scales of Infant and Toddler Development, Third Edition

### Results with adjustment for STH misclassification

Table 7 shows the results from the Bayesian latent class hierarchical models, adjusted for exposure misclassification using the different prior specifications for the sensitivities of the Kato-Katz and direct smear techniques (prior specifications listed in Table 1). Results were dependent on prior specifications for the sensitivities of the diagnostic tests. For *Ascaris* infection and any STH infection, point estimates tended to be further from the null when prior specifications for the sensitivities of the diagnostic tests were lower (i.e. using pessimistic priors). The effect (β (95% CrI)) on cognitive scores between two and five years of age of being infected with *Ascaris* infection one time between one and two years of age compared to never being infected using: a) priors with perfect sensitivities and specificities, b) optimistic priors, c) clinical priors and d) pessimistic priors, was: a) -0.68 (-1.46, 0.10), b) -1.27 (-2.66, 0.12), c) -3.44 (-10.73, 0.19), and d) -4.26 (-9.13, 0.03), respectively. The effect (β (95% CrI) on cognitive scores of being infected with *Ascaris* infection two or three times compared to never being infected using: a) priors with perfect sensitivities and specificities, b) optimistic priors, c) clinical priors and d) pessimistic priors, was: a) -0.63 (-1.60, 0.35), b) -0.99 (-2.23, 0.27), c) -3.08 (-10.32, -0.003), and d) -3.68 (-8.33, -0.40), respectively. Similar effects of the prior specifications were found for the effects of any STH infection. Using the clinical prior specifications, the effects (β (95% CrI)) on cognitive scores of being infected with any STH infection one time and two or three times compared to never being infected were: -4.31 (-10.64, -0.14), and -3.70 (-10.11, -0.11), respectively. Results for *Trichuris* infection were inconclusive but suggest that either no effect or very small effects exist. Similar results were found with respect to the effects on verbal scores (Table 7).

**Table 7:** Results from Bayesian latent class hierarchical models for the effect of STH infection between one and two years of age on child development scores between two and five years of age, adjusted for misclassification of STH infection in preschool children in Iquitos, Peru, September 2011 to July 2016.

	Perfect sensitivity/ specificity	Clinical priors for sensitivity/specificity	Optimistic priors for sensitivity/specificity	Pessimistic priors for sensitivity/specificity
	β (95% CrI)	β (95% CrI)	β (95% CrI)	β (95% CrI)
**1) Outcome: Cognitive score**				
# times found infected with *Ascaris*:				
0	REF	REF	REF	REF
1	-0.68 (-1.46, 0.10)	-3.44 (-10.73, 0.19)	-1.27 (-2.66, 0.12)	-4.26 (-9.13, 0.03)
2–3	-0.63 (-1.60, 0.35)	-3.08 (-10.32, -0.003)	-0.99 (-2.23, 0.27)	-3.68 (-8.33, -0.40)
# times found infected with *Trichuris*:				
0	REF	REF	REF	REF
1	-0.10 (-0.96, 0.75)	-0.08 (-1.23, 1.11)	-0.06 (-1.18, 1.06)	-0.14 (-1.35, 1.11)
2–3	-0.27 (-1.77, 1.23)	0.05 (-2.61, 2.87)	-0.06 (-2.75, 2.73)	0.16 (-2.49, 3.04)
# times found infected with any STH:				
0	REF	REF	REF	REF
1	-0.58 (-1.36, 0.20)	-4.31 (-10.64, -0.14)	-1.42 (-2.86, 0.01)	-5.68 (-9.47, -1.07)
2–3	-0.49 (-1.40, 0.40)	-3.70 (-10.11, -0.11)	-0.94 (-2.21, 0.31)	-4.84 (-8.60, -1.04)
**2) Outcome: Verbal score**				
# times found infected with *Ascaris*:				
0	REF	REF	REF	REF
1	-0.90 (-1.89, 0.08)	-2.81 (-6.05, 0.14)	-1.75 (-3.55, 0.07)	-4.34 (-7.77, 0.002)
2–3	-0.10 (-1.33, 1.13)	-1.41 (-3.88, 0.69)	-0.53 (-2.07, 1.03)	-2.59 (-5.25, 0.31)
# times found infected with *Trichuris*:				
0	REF	REF	REF	REF
1	-0.67 (-1.74, 0.40)	-0.90 (-2.46, 0.61)	-0.93 (-2.43, 0.54)	-0.89 (-2.53, 0.75)
2–3	-0.33 (-2.21, 1.57)	-0.16 (-3.56, 3.17)	-0.003 (-3.46, 3.43)	-0.31 (-3.68, 3.06)
# times found infected with any STH:				
0	REF	REF	REF	REF
1	-0.92 (-1.91, 0.05)	-3.07 (-6.03, -0.24)	-2.17 (-3.97, -0.37)	-4.53 (-7.82, -0.13)
2–3	-0.73 (-1.87, 0.42)	-1.77 (-4.07, 0.30)	-0.95 (-2.54, 0.62)	-2.97 (-5.63, 0.06)

All results are adjusted for maternal education (completed secondary school), cooks using gas, has a toilet with water and connection to public sewage in the home, stunted at one year of age, Bayley-III cognition raw score at one year of age, number of healthy growth visits attended between birth and one year of age and age. All hierarchical models include an individualized intercept term and an individualized slope for age.

## Discussion

Our results, adjusted for exposure misclassification, have shown that infection with *Ascaris* and any STH infection during the critical window of development between one and two years of age can have small effects on cognitive and verbal abilities between two and five years of age. On average, children infected with *Ascaris* between one and two years of age had cognitive and verbal scores between one and four points lower compared to children who were never found infected with *Ascaris*. Children infected with any STH infection between one and two years of age had cognitive and verbal scores between one and six points lower, on average, compared to children who were never found infected between one and two years of age. A lack of precision and general uncertainty regarding the sensitivities of the STH diagnostic techniques led to some uncertainty regarding the exact effect sizes and clinical significance of some results.

As in any study whose results rely on data from imperfect diagnostic techniques, the results are dependent on what has been assumed about the unknown properties of the tests. Here, prior specification about the sensitivities of the STH diagnostic techniques used plays a key role. For example, the results obtained assuming optimistic priors about the test properties are quite different than those obtained using pessimistic priors. For the effect of any STH infection on cognitive scores, the effect of being found infected one time using optimistic priors was -1.42 (-2.86, 0.01) and the same effect using the pessimistic priors was -5.68 (-9.47, -1.07). Due to the lack of a gold standard for diagnosing STH infection, determining the true sensitivities of the diagnostic techniques available is not straight forward. Even limiting our search to studies that used Bayesian methods to account for a lack of a gold standard, we found very different values of sensitivities reported in the literature [27, 31]. The relative uncertainty regarding the true sensitivity values for the STH diagnostic tests was our rationale for using and comparing a range of prior specifications. Our prior specifications were developed based on the research available to date; however, our models suggested that the true sensitivity values may, in fact, be even lower than the pessimistic priors used. This uncertainty regarding the true sensitivity values of the STH diagnostic tests is problematic and limits researchers’ abilities to obtain accurate and informative misclassification-adjusted results for any research question involving STH infections. This is a topic that requires much more research attention.

Despite lacking high precision in estimating effects of STH infections, our results have clinical relevance. If we assume that the true sensitivity values of the STH diagnostic tests are at least as low as the range of the clinical priors used, we found that being infected with *Ascaris* infection two or three times and being infected with any STH infection one time and two or three times during the second year of life are associated with lower cognitive scores between two and five years of age. We also found that being infected with any STH infection one time during the second year of life is associated with lower verbal scores between two and five years of age. These results are consistent with research that suggests that the first two years of life is a particularly critical time period for brain development across the lifespan [2]. In STH-endemic areas, children as young as eight months of age are being found infected [10] and our results suggest that infection during this critical window of development may have long term consequences on brain development. While these results cannot explain the mechanism behind this effect, it has been hypothesized that malnutrition may be responsible, at least in part, for the observed effect. Malnutrition indicators including stunting and anemia have been found to be affected by STH infections and they are also known risk factors for child development [32–35]. The effect of STH infection on child development, therefore, may be due to mediation by malnutrition.

WHO now recommends that children as young as 12 months of age be included in STH control programs [11]; however, large efforts are still required to obtain the goal coverage rates set by WHO of 75% coverage in endemic countries by 2020 [12]. Political motivation to reach preschool children with appropriate STH control programs continues to be a major challenge [36] and this is, in part, due to a lack of rigorous evidence quantifying the disease burden of STH infection in this age group. Our results contribute to the body of research evidence documenting the burden of STH infection in young children as of 12 months of age and support previous research that highlights the importance of targeting preschool children in STH control and prevention approaches [37]. Control strategies should be integrated and, considering the complex relationship between STH infection and malnutrition, combining STH control strategies with nutritional interventions will likely maximize the benefits. Additional research would therefore be valuable to measure the effects of long-term health programs targeted to preschool children that couple STH control with nutrition interventions.

In the last decade considerable attention has been given to the concept of life course epidemiology and several models and structured approaches have been developed to relate exposures over time to a later health outcome [38, 39]. The current analysis involves a combination of two theoretical approaches to quantify the effect of multiple binary exposure measurements collected over time. While previous work has shown that cumulative STH infection during the preschool ages (i.e. between one and five years of age) affects child development [26], this analysis used a critical period framework approach and specifically focussed on the effect of STH infection during the second year of life, a particularly critical window of development. Additionally, an accumulation framework approach was also used by summing indicators of binary variables (i.e. STH infection) over time (i.e. between one and two years of age). We make the assumptions here that STH infection between one and two years of age only is important, irrespective of STH infections at other time points and also make the assumption that the specific timing of infection within the second year of life is unimportant [38].

This study has several strengths and limitations. The longitudinal design allowed us to investigate cumulative STH infections during a particular time window on long term, repeated measures of child development. This allowed us to establish temporality with regard to the timing of the exposure and outcome relationship and allowed us to look at development trajectories as opposed to development scores at one single time point. This is the first study to specifically investigate the effect of STH infection during the second year of life, the most critical period for brain development across the lifespan, on child development. Our adjustment for STH misclassification allowed us to address an important bias due to measurement error. Since no gold standard diagnostic test exists for STH infection, all research involving STH infection is limited by this bias and no previous research on this specific topic has attempted to adjust for this bias. Among the limitations of the research include our proxy measure for amount of time spent STH infected. Because it was unfeasible to collect daily stool specimens and to determine how long each child was infected during their second year of life, we used the number of times found infected at the three scheduled study visits during this period as a proxy measure for amount of time infected. Furthermore, we did not have data regarding STH infection during the first year of life which may be important. The two scales used to measure child development throughout the study (i.e. the Bayley-III and the WPPSI-III) have not been previously validated in this specific population and therefore the test scores may suffer from an unknown amount of measurement error and external comparisons of the scores may be limited. Additionally, it was assumed that the same underlying constructs are measured by the Bayley-III cognitive composite score and the WPPSI-III performance IQ score, and by the Bayley-III language composite score and the WPPSI-III verbal IQ score. Additional limitations include non-verifiable assumptions of our regression models including correct model specification and correct prior specifications for the sensitivity and specificity values used in the analyses adjusted for STH misclassification.

In conclusion, this study has documented associations between *Ascaris* and any STH infection and lower cognitive and verbal scores of child development. A lack of precision led to some uncertainty regarding some of the effect sizes and relative clinical significance of the results. Nonetheless, these results contribute to the body of evidence regarding the burden of STH infection and specifically highlight the importance of STH control and prevention in young children two years of age and younger. While this population group isn’t necessarily the primary target for STH control, we have shown that STH infections at this age may have important and irreversible effects on child development. These results may be generalizable to the 103 LMICs considered endemic for STH infections and provide evidence that can contribute to reducing global inequities in both child development and poverty.

## Supporting information

S1 Checklist STROBE statement. Checklist for reports of observational studies.
(PDF)

S1 File (XLSX)
