# Supplementary material for: A longitudinal cohort study of soil-transmitted helminth infections during the second year of life and associations with reduced long-term cognitive and verbal abilities
Source: PLoS Negl Trop Dis. 2018 Jul 27;12(7):e0006688. doi: 10.1371/journal.pntd.0006688 (PMC6082574; doi:10.1371/journal.pntd.0006688)
Supplement: S1 Checklist — Checklist for reports of observational studies. (PDF) [file pntd.0006688.s001.pdf]

STROBE Statement—checklist of items that should be included in reports of observational studies

	Item No	Recommendation
**Title and abstract**	1	(*a*) Indicate the study's design with a commonly used term in the title or the abstract<br>*-'Cohort study' included in title*
		(*b*) Provide in the abstract an informative and balanced summary of what was done and what was found<br>*- Abstract*
**Introduction**		
Background/rationale	2	Explain the scientific background and rationale for the investigation being reported<br>*- Introduction, paragraph 3:*<br>*"Due to the effects on malnutrition and anemia, STH infections are thought to have an effect on child development. Previous research on this topic has documented associations between STH infections and poor child development; however, the interpretation of this research is limited due to poor research designs [13-21], failure to adjust for important confounding variables [13, 15, 21-23], small sample sizes [15, 22, 24, 25], grouping STH with other parasites in the analyses [18, 23] and inadequate and inappropriate statistical analyses [17-22, 24, 25]. Furthermore, no study has previously looked at the long term effect on development of STH infection specifically during the first two years of life – the most critical period for development across the lifespan."*
Objectives	3	State specific objectives, including any prespecified hypotheses<br>*- Introduction, paragraph 3:*<br>*"The objective of the current research, therefore, was to evaluate the long-term effect of STH infection between one and two years of age on repeated measures of child development between two and five years of age."*
**Methods**		
Study design	4	Present key elements of study design early in the paper<br>*- Methods, paragraph 1:*<br>*"Briefly, a longitudinal cohort study was conducted in the rural and peri-urban areas surrounding Iquitos, Peru between September 2011 and July 2016. A total of 1,760 children were recruited at 12 months of age and followed-up at 18 months and at two, three, four and five years of age."*
Setting	5	Describe the setting, locations, and relevant dates, including periods of recruitment, exposure, follow-up, and data collection<br>*- Methods, paragraph 4 (see item 4)*
Participants	6	(*a*) *Cohort study*—Give the eligibility criteria, and the sources and methods of selection of participants. Describe methods of follow-up<br>*- Methods, paragraph 1:*<br>*"During recruitment, a sampling frame for the study was obtained from participating health centre records and from a door-to-door census conducted in the study area by the research team before initiation of the study. The study population consisted of eligible children living in the catchment areas of the twelve major health centres serving the three rural/peri-urban communities surrounding Iquitos (i.e. Nanay, Belén and San Juan), who, during study recruitment, were between 12 and 14 months of age. Study inclusion criteria included: 1) children between 12 and 14 months of age at recruitment; 2) children attending one of the participating health centres for their 12-month routine healthy growth and development visit (note that the parent/guardian of any child who was identified as a potential participant from the sampling frame but who did not attend his/her routine visit at 12 months of age was contacted at home by a research assistant and encouraged to attend); 3) children who were not consulting medical advice for a suspected STH infection; 4) children who had not been dewormed in the six months prior to their recruitment into the study; and 5) children who did not have any serious congenital or chronic medical condition. Study exclusion criteria included: 1) children who lived outside of the*

		identified study area; 2) children whose family planned to move outside of the study area in the year following recruitment; 3) children whose parents did not consent to participate in the study."
		(*b*) *Cohort study*—For matched studies, give matching criteria and number of exposed and unexposed<br>*-Not applicable*
Variables	7	Clearly define all outcomes, exposures, predictors, potential confounders, and effect modifiers. Give diagnostic criteria, if applicable<br>*- Methods, paragraphs 2-6:*<br>*"Exposure Ascertainment... Outcome ascertainment... Measurement of covariates"*
Data sources/ measurement	8*	For each variable of interest, give sources of data and details of methods of assessment (measurement). Describe comparability of assessment methods if there is more than one group<br>*- Methods, paragraphs 2-6:*<br>*"Exposure ascertainment... Outcome ascertainment... Measurement of covariates"*
Bias	9	Describe any efforts to address potential sources of bias<br>*1) To address confounding (Statistical analyses, paragraph 3): "Both univariable and multivariable regression models are presented. The covariates included in the final models were chosen based on theoretical knowledge (i.e. confounding variables that are thought to be associated with both the exposure and outcome of interest without being mediators of this relationship) and by statistical criteria..."*<br>*2) To address measurement error (Statistical analyses, paragraph 6): "To address exposure misclassification due to imperfect sensitivities and specificities of the diagnostic tests used to measure STH infection, Bayesian latent class hierarchical regression models were used…"*
Study size	10	Explain how the study size was arrived at<br>*-Sample size:*<br>*"A sample size of 880 children with four measures of the outcome per child (i.e. at 2, 3, 4 and 5 years of age) was available for analysis. The primary outcome, cognitive score, was compared between children who were never found STH infected to children who were found infected one time and two or three times. No estimate of the ICC for repeated measures of cognitive scores of children between two and five years of age was found in the literature. Therefore, based on preliminary data, an ICC for cognitive scores was estimated to be 0.2. This corresponds to a design effect of 1.6 (design effect = 1 + (# observations per child – 1) × ICC). Assuming that the repeated observations were independent, a total sample size of 3,520 would be available (880 × 4). Taking the correlation between repeated measures into account, the effective sample size is 2,200 (3,520 / 1.6). Based on expert opinion in child development, a difference in mean scores of 5 points (i.e. 1/3 of a standard deviation) was considered the minimum clinically significant effect size. Assuming that the standard deviation of cognitive scores is 15, and having known from preliminary data that 43% of the population is unexposed (never STH-infected) and that 36% and 21% of the population were found STH-infected one, and two or three times, respectively (based on preliminary data), an effective sample size of 2,200 would be able to detect a difference of 5 points in cognitive scores between never infected and infected once, and between never infected and infected two or three times, with total 95% confidence interval widths of 2.84 and 3.34, respectively. Therefore, the sample size provided sufficient precision for the planned comparisons."*
Quantitative variables	11	Explain how quantitative variables were handled in the analyses. If applicable, describe which groupings were chosen and why<br>*- Statistical analysis, paragraph 2:*<br>*"The exposure was categorized into found infected zero times, one time, two times and three times. Due to the low number of children who were found infected at all three time points between one and two years of age, the categories for being found infected two and three times were combined."*
Statistical methods	12	(*a*) Describe all statistical methods, including those used to control for confounding<br>*- Statistical analysis section, paragraphs 2-6:*

		"Hierarchical linear regression models were used to investigate the effect of the number of times a child was detected STH infected between one and two years of age on repeated measures of development scores at two, three, four and five years of age… Both univariable and multivariable regression models are presented… Missing exposure and outcome data were imputed using multiple imputation… To address exposure misclassification due to imperfect sensitivities and specificities of the diagnostic tests used to measure STH infection, Bayesian latent class hierarchical regression models were used…"
		(*b*) Describe any methods used to examine subgroups and interactions<br>*-Not applicable*
		(*c*) Explain how missing data were addressed<br>*- Statistical analysis, paragraph 5:*<br>*"Missing exposure and outcome data were imputed using multiple imputation. No covariate had missing data. Multinomial regression models were used as the imputation models for cumulative STH infections and linear regression models were used as the imputation models for development scores. All covariates included in the outcome models were also included in the imputation models as well as other relevant covariates, with complete data, that predicted the missing data, as appropriate."*
		(*d*) *Cohort study*—If applicable, explain how loss to follow-up was addressed<br>*-Missing data (due to loss to follow-up): Statistical analysis, paragraph 5*
		(*e*) Describe any sensitivity analyses<br>*- Statistical analysis, paragraphs 6-7 :*<br>*"To address exposure misclassification due to imperfect sensitivities and specificities of the diagnostic tests used to measure STH infection, Bayesian latent class hierarchical regression models were used. This method was particularly useful in this context because it allows for individual variation in sensitivity and specificity values (here, due to the fact that, while the majority of stool specimens were analyzed with the Kato-Katz technique, some stool specimens were analyzed with the direct smear technique) and because a gold standard diagnostic technique for STH infection does not exist and therefore the sensitivity and specificity values are not exactly known. This method has been described in detail and used previously [30]. Briefly, within the latent class analysis, three separate models were specified: 1) The outcome model is a hierarchical linear regression that models child development scores conditional on latent STH infection (i.e. the true, unmeasured exposure) and the confounding variables mentioned previously. An individualized intercept and slope for age was included according to the previous description. The main effect estimate of interest is the effect of latent STH infection on child development that is estimated in this model.; 2) The exposure models are logistic regressions that model true latent STH infection status at 12, 18 and 24 months of age, conditional on covariates that predict species-specific STH infection. These covariates were chosen based on Bayesian information criterion (BIC) criterion. The exposure models allow for differences in the probabilities of being STH-infected between various groups of children to be accounted for; and 3) The misclassification models predict the measured STH infection status at each of the three time points according to the true latent STH infection status at each time point and the sensitivity and specificity values of the diagnostic technique used (i.e. Kato-Katz technique or direct smear technique) at each time point…"*
**Results**		
Participants	13*	(a) Report numbers of individuals at each stage of study—eg numbers potentially eligible, examined for eligibility, confirmed eligible, included in the study, completing follow-up, and analysed<br>*- Figure 1 & Results, paragraph 1*
		(b) Give reasons for non-participation at each stage<br>*- Figure 1 & Results, paragraph 1*
		(c) Consider use of a flow diagram<br>*-Figure 1*

Descriptive data	14*	(a) Give characteristics of study participants (eg demographic, clinical, social) and information on exposures and potential confounders<br>*- Tables 2-3 & Results, paragraphs 1-2*
		(b) Indicate number of participants with missing data for each variable of interest<br>*-Figure 1 & Results, paragraph 1:*<br>*"Missing STH infection data were present for four children at 18 months of age and for four children at 24 months of age due to loss to follow-up. Number of times found STH infected between one and two years of age was therefore missing for a total of eight children (< 1%). Development scores were missing for a total of four children at the two years of age visit due to loss to follow-up. At the three years of age visit, development scores were missing for two children due to a protocol violation (two children who should have been administered the Bayley-III were not, due to an error by the research assistant). At the four years of age visit, development scores were missing for a total of 63 children (7%): 46 due to loss to follow-up, 15 due to invalid WPPSI-III measurements (WPPSI-III measurements are considered invalid if a participant scores 0 on two or more performance subscales and/or verbal subscales) and two due to protocol violations. At the five year visit, development scores were missing for a total of 99 children (11%): 85 due to loss to follow-up and 14 due to invalid WPPSI-III measurements."*
		(c) *Cohort study*—Summarise follow-up time (eg, average and total amount)<br>*-Figure 1 & Results, paragraph 1*
Outcome data	15*	*Cohort study*—Report numbers of outcome events or summary measures over time<br>*-Table 4 & Results, paragraph 2:*<br>*"Raw, scaled and composite development scores from the Bayley-III and the WPPSI-III between one and five years of age are presented in Table 4. Overall, composite scores decreased over time with the highest scores obtained at the baseline visit and the lowest scores obtained at the final, five years of age visit"*
		Case-control study—Report numbers in each exposure category, or summary measures of exposure
		Cross-sectional study—Report numbers of outcome events or summary measures
Main results	16	(*a*) Give unadjusted estimates and, if applicable, confounder-adjusted estimates and their precision (eg, 95% confidence interval). Make clear which confounders were adjusted for and why they were included<br>*- Unadjusted and adjusted estimates: Tables 5-6.*<br>*-Justification of confounders included: Statistical Analyses, paragraph 3:*<br>*"The covariates included in the final models were chosen based on theoretical knowledge (i.e. confounding variables that are thought to be associated with both the exposure and outcome of interest without being mediators of this relationship) and by statistical criteria. Baseline variables considered as potential confounders included: socioeconomic status (i.e. residential district, urban/rural status, mother's marital status (i.e. married/common-law vs single), maternal education (i.e. secondary education completed), mother employed, father or mother's partner employed, number of people living in the home, house material, cooks using gas, presence of electricity in the home, working radio ownership, working television ownership, water source, has a toilet with water and connection to public sewage in the home, and household income); sex; healthcare seeking behavior (i.e. number of healthy growth visits attended from birth to one year of age and vaccines up to date at baseline); hygiene (i.e. number of baths per day and use of soap for bathing); hospitalizations since birth; anthropometry/malnutrition (i.e. stunted, underweight, wasted, birth weight); baseline development scores (i.e. Bayley-III cognitive raw score, Bayley-III receptive language raw score, Bayley-III expressive language raw score and, Bayley-III fine motor raw score); and breastfeeding (i.e. exclusively breastfed to six months and continued breastfeeding at one year). To perform model selection, univariable hierarchical linear regression models were used to determine if each variable was associated with the outcome variables and univariable multinomial regression models were used to determine if each variable was associated with the exposure variables. Correlations and 2 x 2 tables were also used to observe relationships between the confounding variables. The final presented models include confounding variables that are associated with both the exposure and outcome and that affected the association between the exposure and outcome of interest. These include socioeconomic status (i.e. maternal education, cooks using gas, has a toilet with water and connection to public sewage in the home), baseline nutritional status (i.e. stunted), use of health care (i.e.*

		number of healthy growth visits attended from birth to one year of age), baseline development scores (i.e. Bayley-III cognition raw scores) and age."
		(*b*) Report category boundaries when continuous variables were categorized<br>*-Not applicable*
		(*c*) If relevant, consider translating estimates of relative risk into absolute risk for a meaningful time period<br>*-Not applicable (outcome is continuous)*
Other analyses	17	Report other analyses done—eg analyses of subgroups and interactions, and sensitivity analyses<br>*-Table 7 & Results, paragraph 4*
**Discussion**		
Key results	18	Summarise key results with reference to study objectives<br>*-Discussion, paragraph 1:*<br>*"Our results, adjusted for exposure misclassification, have shown that infection with Ascaris and any STH infection during the critical window of development between one and two years of age can have small effects on cognitive and verbal abilities between two and five years of age. On average, children infected with Ascaris between one and two years of age had cognitive and verbal scores between one and four points lower compared to children who were never found infected with Ascaris. Children infected with any STH infection between one and two years of age had cognitive and verbal scores between one and six points lower, on average, compared to children who were never found infected between one and two years of age."*
Limitations	19	Discuss limitations of the study, taking into account sources of potential bias or imprecision. Discuss both direction and magnitude of any potential bias<br>*-Discussion, paragraph 6:*<br>*"Among the limitations of the research include our proxy measure for amount of time spent STH infected. Because it was unfeasible to collect daily stool specimens and to determine how long each child was infected during their second year of life, we used the number of times found infected at the three scheduled study visits during this period as a proxy measure for amount of time infected. Furthermore, we did not have data regarding STH infection during the first year of life which may be important. The two scales used to measure child development throughout the study (i.e. the Bayley-III and the WPPSI-III) have not been previously validated in this specific population and therefore the test scores may suffer from an unknown amount of measurement error and external comparisons of the scores may be limited. Additionally, it was assumed that the same underlying constructs are measured by the Bayley-III cognitive composite score and the WPPSI-III performance IQ score, and by the Bayley-III language composite score and the WPPSI-III verbal IQ score. Additional limitations include non-verifiable assumptions of our regression models including correct model specification and correct prior specifications for the sensitivity and specificity values used in the analyses adjusted for STH misclassification."*
Interpretation	20	Give a cautious overall interpretation of results considering objectives, limitations, multiplicity of analyses, results from similar studies, and other relevant evidence<br>*- Discussion, paragraph 7:*<br>*"In conclusion, this study has documented associations between Ascaris and any STH infection and lower cognitive and verbal scores of child development. A lack of precision led to some uncertainty regarding some of the effect sizes and relative clinical significance of the results. Nonetheless, these results contribute to the body of evidence regarding the burden of STH infection and specifically highlight the importance of STH control and prevention in young children two years of age and younger. While this population group isn't necessarily the primary target for STH control, we have shown that STH infections at this age may have important and irreversible effects on child development."*
Generalisability	21	Discuss the generalisability (external validity) of the study results<br>*-Discussion, paragraph 7:*<br>*"These results may be generalizable to the 103 LMICs considered endemic for STH infections and provide evidence that can contribute to reducing global inequities in both child development and poverty."*
**Other information**		

Funding	22	Give the source of funding and the role of the funders for the present study and, if applicable, for the original study on which the present article is based<br>*-Funding statement*

*Give information separately for cases and controls in case-control studies and, if applicable, for exposed and unexposed groups in cohort and cross-sectional studies.

Note: An Explanation and Elaboration article discusses each checklist item and gives methodological background and published examples of transparent reporting. The STROBE checklist is best used in conjunction with this article (freely available on the Web sites of PLoS Medicine at http://www.plosmedicine.org/, Annals of Internal Medicine at http://www.annals.org/, and Epidemiology at http://www.epidem.com/). Information on the STROBE Initiative is available at www.strobe-statement.org.
